# GAILS: an effective multi-object job shop scheduler based on genetic algorithm and iterative local search

**DOI:** 10.1038/s41598-024-51778-1

**Published:** 2024-01-24

**Authors:** Xiaorui Shao, Fuladi Shubhendu Kshitij, Chang Soo Kim

**Affiliations:** 1 Industrial Science Technology Research Center, Pukyong National University, Busan, 608737 Korea; 2 Department of Information Systems, Pukyong National University, Busan, 608737 Korea

**Keywords:** Energy science and technology, Engineering

## Abstract

The job shop scheduling problem (JSSP) is critical for building one smart factory regarding resource management, effective production, and intelligent supply. However, it is still very challenging due to the complex production environment. Besides, most current research only focuses on classical JSSP, while flexible JSSP (FJSSP) is more usual. This article proposes an effective method, GAILS, to deal with JSSP and FJSSP based on genetic algorithm (GA) and iterative local search (ILS). GA is used to find the approximate global solution for the JSSP instance. Each instance was encoded into machine and subtask sequences. The corresponding machine and subtasks chromosome could be obtained through serval-time gene selection, crossover, and mutation. Moreover, multi-objects, including makespan, average utilization ratio, and maximum loading, are used to choose the best chromosome to guide ILS to explore the best local path. Therefore, the proposed method has an excellent search capacity and could balance globality and diversity. To verify the proposed method's effectiveness, the authors compared it with some state-of-the-art methods on sixty-six public JSSP and FJSSP instances. The comparative analysis confirmed the proposed method's effectiveness for classical JSSP and FJSSP in makespan, average utilization ratio, and maximum loading. Primarily, it obtains optimal-like solutions for several instances and outperforms others in most instances.

## Introduction

With the continuous development of global economics, modern industry is more and more complex^1,2^, causing a new challenge for implementing smart factories^3^. Job shop scheduling plays a core role in the process of building one smart factory, which has extracted more attention^4,5^.

The current methods for solving JSSP consist of exact and approximation methods. The exact methods that can obtain optimal solutions while it is very time-consuming and resource-consuming^6^, especially when meeting one large-scale JSSP instance. Therefore, most current methods mainly focus on approximated methods, including the simplest dispatching rules (DRs) and artificial intelligence (AI)^7^. Among these two methods, DRs such as first in first out (FIFO), shortest processing time (SPT), and earliest completion time first (ECTF) are the most straightforward and simplest but are far from optimal solutions^8^. On the contrary, the AI-based methods extract the hidden information from the JSSP instances to construct the corresponding rules more accurately within a tolerable time, which has been mainstream for solving JSSP. It mainly consists of the artificial immune algorithm^9,10^, genetic algorithm (GA)^11^, swarm intelligence (SI)^12^, local search (LS) algorithms, and network-based approaches^13–15^, etc. GA and neural network-based (NN) methods have extracted more attention.

GA is one global searching algorithm with parallelism, robustness, wide applicability, and interpretability^16^, which has been widely used for solving JSSP-like optimization problems. For instance, Omar et al. applied one improved GA to solve JSSP^17^, in which they initialed the population with some well-known DRs rather than a random solution. Teekeng et al. proposed one improved GA for solving FJSSP by changing three operators: selection, crossover, and mutation^18^. Mohamed^19^ proposed a new GA based on the island model for solving JSSP using a migration selection mechanism to evaluate and select the genes, whose effectiveness is demonstrated on 52 public JSSP instances. Li et al.^7^ applied GA to search the global solution first, and then Tabu search was used to find the best local solution for solving both JSSP and FJSSP. Sun et al.^20^ applied GA with variable neighborhood search for solving FJSSP. Besides, similar to the idea of GA, Lu et al.^21^ proposed an improved iterated greedy (IIG) algorithm to solve the distributed hybrid flow shop scheduling problem. They constructed three operators to search for the global path, and one LS algorithm consisting of four neighborhood structures is designed to find its best local path. Moreover, GA-based algorithms are developed to solve other JSSP-like problems, including timetabling scheduling^22,23^, traveling salesman problem (TSP)^24^, and network parameter optimization^25,26^.

The NN-based methods have two branches: traditional and deep reinforcement learning (DRL). Traditional learning methods for solving JSSP treat it as one sub-classification problem. It uses other well-known algorithms, such as DRs and GA, to obtain corresponding labels (the priority for each sub-task) and simultaneously calculates the corresponding statuses used to train the model. The trained model is used to predict the priority of each sub-task, which is converted into a JSSP pattern at the end. E. g., Weckman et al.^13^ are the first to apply NN for solving JSSP. They utilized GA to solve 6 $\times$ 6 JSSP instances first, and then one NN with three hidden layers was adopted to predict each subtask's priority by inputting twelve features. The comparative results on ft06^27^ indicated that the NN-based method outperforms attribute-oriented induction (AOI) and SPT but is still far from GA.

Recent deep learning (DL) technology has achieved great success in many fields, such as image classification^28^, fault diagnosis^29^, and medical healthcare^30^, due to its powerful feature extraction capacity. Also, DL has extracted attention in the field of JSSP. For instance, Zang et al.^31^ applied a convolutional neural network (CNN) to extract the hidden features from ten input features and their transformations corresponding to the sub-task generated from GA. Shao et al.^14^ employed GA to generate training samples and long-short-term memory (LSTM) with K-means to mine key hidden features for solving JSSP. Besides, Kim et al.^8^ applied multi-level CNN (ML-CNN) to find the approximate global path and applied ILS to explore the best local solution for solving JSSP.

DRL^32^ describes one JSSP as a Markov decision process (MDP), in which the DL part extracts rich hidden features that reflect the current dynamic production environmental state $s$ to predict the corresponding reward $r$. The RL part records a pair of actions and rewards. Significantly, the current state will be transformed into a new state ${s}^{{{\prime}}}$ by doing action $a$ and returning a reward $r$. The DRL arranges all sub-tasks in one JSSP instance by maximizing the total reward. E.g., Ye et al.^33^ utilized DRL for resource scheduling, which utilizes one-dimension (1-D) CNN to extract the hidden dynamic features. Lin et al.^34^ proposed a novel multi-class DRL-based method, deep Q network (DQN), to select the rule for each machine to arrange a corresponding job, in which six DRs are utilized. Considering the shortcoming of a single DQN that predicts and evaluates the action using the same model, double DQN (DDQN) is applied to solve JSSP within eighteen DRs^4^. Besides, Liu et al.^35^ combined actor-critic with reinforcement learning (ACRL) to solve JSSP, and the comparative results in terms of makespan on some public JSSP instances indicated its effectiveness; and a graph network is combined with Q-learning to solve JSSP in traffic management^36^.

Although the abovementioned methods have obtained good performance, they still have some limitations, as described in Table 1. The exact method is the most accurate but cannot deal with large-scale JSSP instances and is time-consuming; DRs are simplest but not adequate^37^; The GA-based method could obtain a near-optimal solution for JSSP instances due to its good global exploring ability, but it lacks some of the local searching ability; On the contrary, LS lacks global exploring ability; Traditional learning methods could solve JSSP fast but highly depends on other algorithms; DRL-based methods are effective and intelligent, but how to design dynamic input nodes and reward function still need to think more.

**Table 1:** The description of each method for JSSP.

	Method	Advantages	Limitations
	Exact	Optimal	Cannot deal with large-scale instances, time-consumption
Approximation	DRs	Simple	Not effective
	GA	Near-optimal, Good global exploring ability	Lacks local searching ability
	LS	Good local searching ability	Lacks global exploring ability
	AI-based		
	Traditional learning	Fast	Highly relies on other algorithms
	DRL	Effective and intelligent	Hard to select input nodes and reward function

Table 1 shows that no algorithm can handle all JSSP-like optimization problems well. Besides, most current methods only focused on JSSP or FJSSP, except for^7^. They evaluated the proposed method in makespan, which could not satisfy human beings' needs. Motivated by those, this article presents one effective scheduler, GAILS, to solve multi-objective JSSP and FJSSP. In which an improved GA based on reference^7^ is designed to find the approximate global solution for the JSSP and FJSSP instances. The initial chromosome of machine and subtask sequences are randomly set first. Then, three operators, including selection, crossover, and mutation, are designed to explore the global genes for each sequence. Moreover, the multi-object fitness function is designed by combining makespan, average utilization ratio, and maximum loading to choose the best global chromosome to guide ILS to explore the best local path. The reason for choosing ILS rather than other local searching algorithms is to find its best local path because ILS can ensure the solution's diversity by adjusting different perturbation strategies^38^. Powered by GA, ILS, and multi-object fitness function, the proposed method has an excellent searching capacity and could balance globality and diversity for solving JSSP and FJSSP.

The main contributions of this article are summarized as follows:

1. An improved GA is proposed to find the global solution of JSSP and FJSSP instances using three new operators: selection, crossover, and mutation. The comparative results showed that the improved GA is more effective than traditional GA.
2. The ILS is applied to explore the optimal local path from GA obtained global path, which ensures the solution is near optimal.
3. A multi-object fitness function that contains makespan, average utilization rate (AUR), and maximum loading (ML) is designed to select the best global and local path, which could be easily extended to other optimization algorithms. Besides, it can easily attach personal goals for different production statutes by adjusting their order in the fitness function.
4. Based on the good design of GA, ILS, and multi-object fitness function, the proposed method has an excellent searching capacity. It could balance globality and diversity for solving JSSP and FJSSP. The Comparative analysis based on sixty public instances confirmed its effectiveness in terms of makespan, average utilization rate, and maximum loading. In addition, the effectiveness of GA and ILS has been analyzed in the proposed method.

The rest of this article is arranged as follows. Section “Problem definition” defines the JSSP and FJSSP. Section “The proposed methods” presents the proposed method in detail. Section “Experimental verification” performs the experimental verification based on public JSSP and FJSSP instances. In section “Discussion”, we discuss the proposed method in depth. Section “Conclusions” concludes this article.

## Problem definition

JSSP aims at arranging $n$ jobs ${\mathbb{J}}=\{{J}_{1},{ J}_{2},\dots ,{ J}_{i}\dots ,{ J}_{n}\}$ to be processed by $m$ machines ${\mathbb{M}}=\{{M}_{1},{ M}_{2},\dots ,{ M}_{j},\dots ,{ M}_{m}\}$ with satisfactory metrics such as makespan, AUR, and ML. Where each job ${J}_{i}$ consists of ${n}_{i}$ operations ${\mathbb{O}}=\{{O}_{i,1},{O}_{i,2},{O}_{i,3},\dots ,{O}_{i,{\text{k}}},\dots ,{O}_{i,{n}_{i}}|\}$. Each operation ${O}_{i,k}(i={1,2},3,\dots ,n;k={1,2},3,\dots ,{ n}_{i})$ needs to be processed by a functional machine ${M}_{j}$ within a particular time ${T}_{i,j}\in {\mathbb{T}}=\{{T}_{i,1},{ T}_{i,2},{ T}_{i,3},\dots ,{{ T}_{i,k},\dots ,T}_{{ i, n}_{i}}\}$. Notice that one operation in the JSSP instance can only be processed once by one certain machine.

Unlike classical JSSP, FJSSP needs to determine both a job operation and the corresponding machine to execute the selected operation to attach good criteria. That is, each operation ${O}_{i,j}\in {\mathbb{O}}$ needs to be processed by a machine ${M}_{i,j,}$ out of a given set ${\mathbb{M}}_{s}$ $\in {\mathbb{M}}$. From the definition of JSSP and FJSSP, we know that FJSSP is one complex kind of JSSP. Generally, both JSSP and FJSSP pursue finding the minimized makespan, defined as (1). Where ${C}_{i,k}$ is the completion time of $k$ th operation for job $i$, and ${C}_{max}$ is the maximum completion time for all operations. Besides, this manuscript aims at developing one accurate algorithm to attach multiple goals, including AUR, and ML, as shown in (2) for AUR, and (3) for ML. Where $En{d}_{i}={{\text{max}}\left({C}_{i, j}\right)}_{{m}_{i}}$ is the ending time of machine ${m}_{i}$, and $idl{e}_{i}$ is the idle time of ${m}_{i}$.

1 $${{\text{C}}}_{{max}}=max({C}_{i,k}|i={1,2},..,n;k={1,2},..,{n}_{i})$$

2 $$AUR=\frac{\sum_{i=0}^{m}(En{d}_{i}-idl{e}_{i})/En{d}_{i}}{m}$$

3 $$ML=Max\left(En{d}_{i}-idl{e}_{i}\right),where i={1,2},3,\dots ,m.$$

To simplify the problem, we define four constraints for both JSSP and FJSSP, as follows:

4 $$\left\{\begin{array}{c}{C}_{i,k}={T}_{i,k} \left(i,k\right)\in O and k=1 4.a\\ {C}_{i,k+1}-{C}_{i,k}\ge {T}_{i,k+1} if \left(i,j\right)\to \left(i,k\right), k\in {\mathbb{M}}^{j} 4.b\\ {C}_{i,k}-{C}_{i,j}\ge {T}_{i,k} Or {C}_{i,j}-{T}_{i,k}\ge {C}_{i,k}, \left(i,j\right),\left(i,k\right)\subseteq O,j\ne k 4.c\\ {C}_{i,k+1}-{C}_{i,k}={T}_{i,k+1} 4.d\end{array}\right.$$

***Constraint 1:*** Starting time constraint, Eq. 4 (a) indicated that the completing time ${C}_{i,k}$ equals its operation time ${T}_{i,1}$, which indicates that all jobs start from zero.

***Constraint 2***: Operation order constraint, Eq. 4 (b) indicated that one job ${J}_{i}$ has specific orders $\{{O}_{i,1},{ O}_{i,2},{ O}_{i,3},\dots ,{O}_{{ Oi, n}_{i}}\}$ to execute at corresponding machine $\{{M}_{i,1},{ M}_{i,2},{ M}_{i,3},\dots ,{ M}_{{ i, n}_{i}}\}$ in a certain time $\{{T}_{i,1},{ T}_{i,2},{ T}_{i,3},\dots ,{{ T}_{i,n}}_{i}\}\in {\mathbb{T}}$ for JSSP since the difference between ${C}_{i,k+1}$ and ${C}_{i,k}$ is greater or equal to the operation time ${T}_{i,k+1}$. For FJSSP, the only difference is that each sub-operation ${O}_{i,k}$ requires selecting the best machine ${M}_{i,j}$ from a given machine set ${\mathbb{M}}_{{\text{s}}}$ to execute within the corresponding time duration. That is, the next sub-operation will start after completing the previous sub-operation.

***Constraint 3***: Resource constraint, Eq. 4 (c) indicated that the machine ${M}_{j}$ can only process one given sub-operation ${O}_{i,j}$ at once. Where different sub-operations for the $i$ th job’s completing time difference ${C}_{i,k}-{C}_{i,j}$ should be greater or equal to the $k$ th sub-operation time ${T}_{i,k}$.

***Constraint 4***: Eq. 4 (d) showed that we do not consider the set-up and transmission times during scheduling.

## The proposed methods

**Figure 1:**
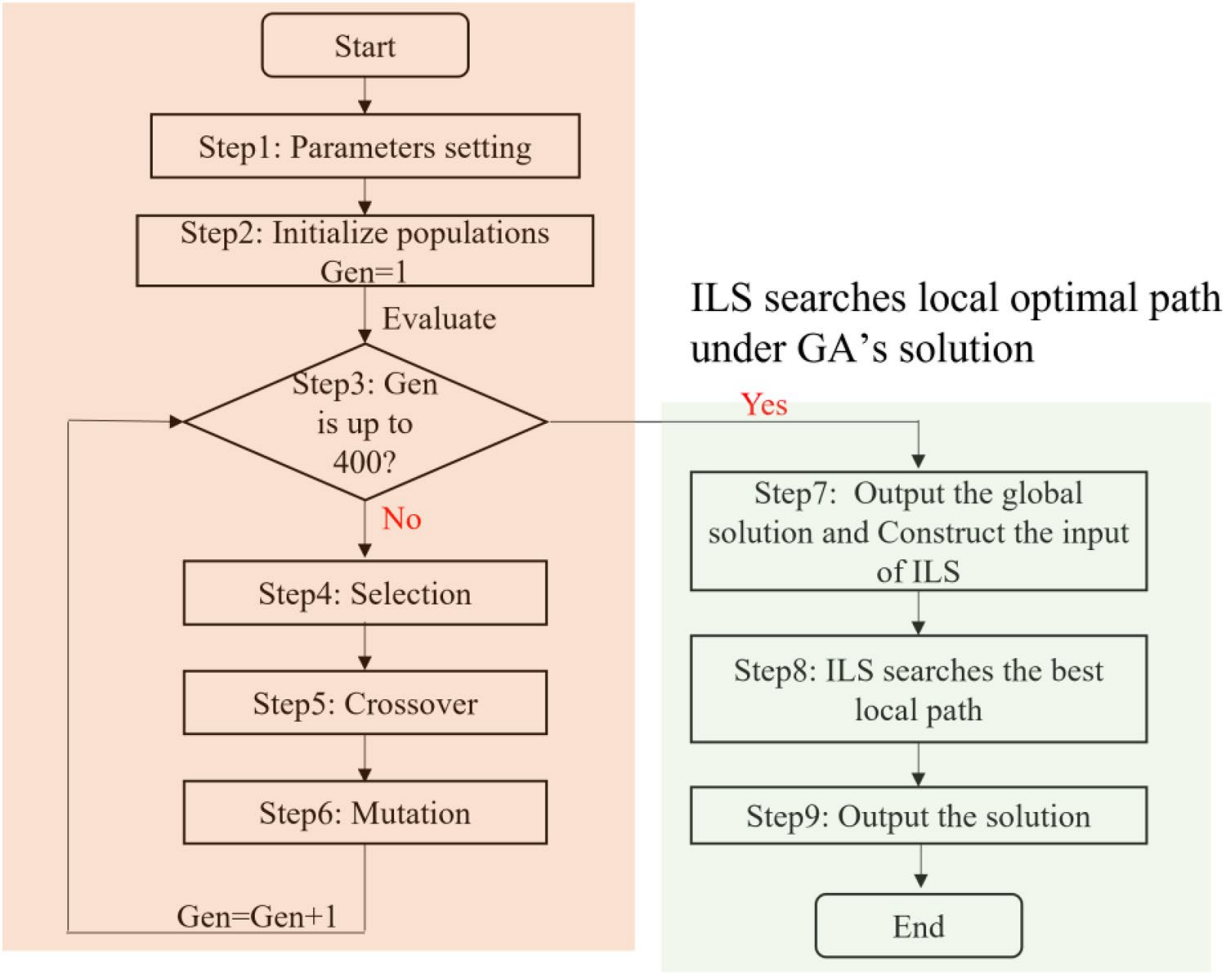
The workflow of the proposed GAILS for solving JSSP and FJSSP.

The proposed method consists of two parts: GA finds the global path (step 1 to step 6), and ILS explores the optimal local path (step 7 to step 9), whose workflow is shown in Fig. 1. The overall procedure of the proposed method is described in the subsequent sections.

***Step 1:*** Setting the parameters of the proposed method, including max generation $Gen$, population size $Po{p}_{size}$, crossover ratio $Cr$, and mutation ratio $Mr$. Setting a bigger $Gen$ and $Po{p}_{size}$ may receive more satisfactory results, but they require massive resources to find the global path. Considering both performance and time costing, we set $Gen$ and $Po{p}_{size}$ as 400, which is similar to the work of Gao^7^. Moreover, the relatively bigger crossover and smaller mutation ratios can ensure gene diversity while simultaneously preserving excellent genes to optimize the algorithm. Thus, we set the crossover ratio $Cr$ and mutation ratio $Mr$ as 0.8 and 0.1, respectively.

***Step 2:*** Initialize the population $Pop$. Each individual has an operation sequence (OS) and a machine sequence (MS). Both OS and MS are equal to $\sum_{i=0}^{n}{O}_{i{,n}_{i}}$, generated by algorithm 1. Running algorithm 1 $Po{p}_{size}$ times to initialize populations. The gene in OS represents the processing order of each job's operations on a specific machine over the occurring orders. The gene in MS represents the corresponding machine over the job and operation.

**Algorithm 1:**
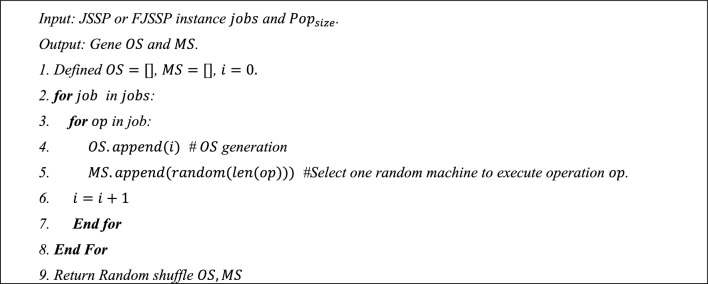
OS and MS Encoding Methods

**Figure 2:**
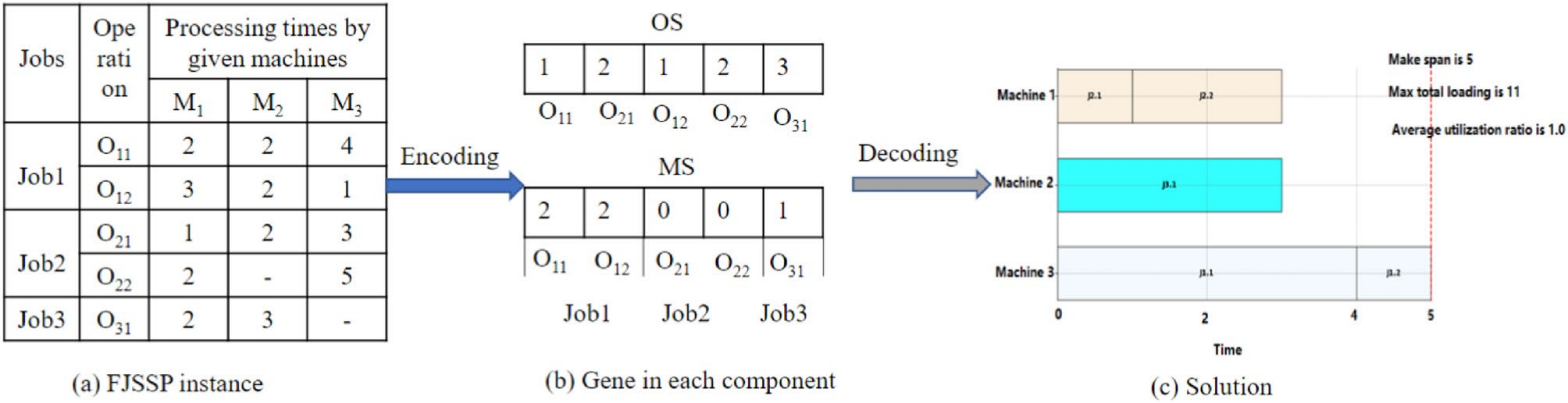
One example of a 3 $\times$ 3 FJSSP instance.

One $3\times 3$ FJSSP instance is used to explain, as shown in Fig. 2. The code of OS = {1,2,1,2,3} corresponding to operation ${\{{\text{O}}}_{11}, {{\text{O}}}_{21},{{\text{O}}}_{12},{{\text{O}}}_{22},{{\text{O}}}_{31}\}$; And MS = {2,2,0,0,1} responds ${\{{\text{O}}}_{11},{{\text{O}}}_{12},{{\text{O}}}_{21},{{\text{O}}}_{21},{{\text{O}}}_{22},{{\text{O}}}_{23}\}$ will be processed by machine {3,3,1,1,2}, respectively. After obtaining the final chromosomes, the decoding method generates the FJSSP pattern in step 9. The JSSP only needs to update OS while all elements of MS are equal to 0 since it only requires one machine for one sub-operation.

***Step 3***: Evaluate the population. If $Gen$ is up to 400, it will output the global solution and feed it into ILS in step 7. Else, go to step 4. The evaluation metric is the combination of {$Makespan$,$AUR, ML$}. The makespan is defined as (1), AUR and ML are expressed as (2) and (3). Noticing that the traditional JSSP instance's operation-machine pair is already given and cannot change during the scheduling. Therefore, the ML is the same for all methods in JSSP but is different in FJSSP. As a result, the proposed method will first evaluate the population by ${C}_{max}$; if several solutions have the same ${C}_{max}$, then check $AUR$ for JSSP and check AUR and $ML$ for FJSSP, respectively.

***Step 4***: Selection operator. The proposed method combined elitist and tournament selection methods as selection operators. Mainly, elitist selection copies 5% of individuals from the original population $Pop$ as a part of the new population ${\text{newPop}}$. The rest 95% of the new population is generated from the tournament selection algorithm. The tournament selection algorithm sets ${\text{k}}=2$ to select the rest of the individuals. That is, two individuals are evaluated using the fitness function $Best=\{{C}_{m}$*,*$AUR, ML\}$, and the best one will be selected and added to the new population. The whole algorithm, as described in Algorithm 2.

**Algorithm 2:**
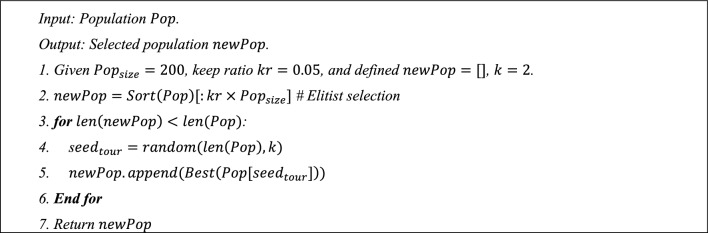
Selection algorithm

***Step 5***: Crossover operator. The proposed method adopted precedence operation crossover (POX) and job-based crossover (JBX) for OS string. Each selected 50% randomly to crossover the OS string and adopted a two-point crossover for the MS string, which is identical to^7^.

**Figure 3:**
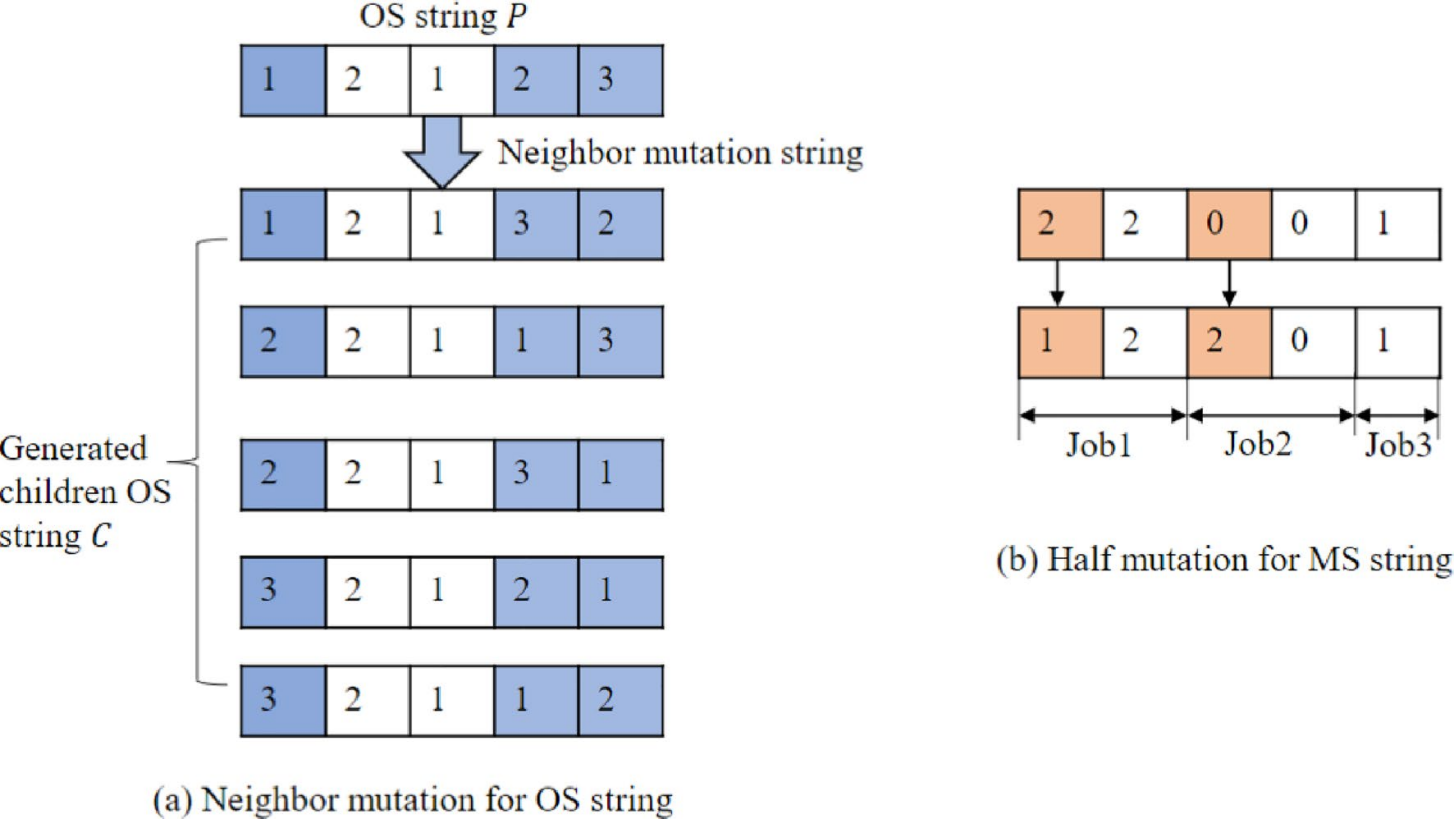
Mutation operator for OS and MS.

***Step 6***: Mutation operator. The proposed method applies neighbor mutation for 15% of the OS string. The process of neighbor mutation is described in the following steps and Fig. 3 (a).

1) Randomly select three different elements in parent $P$ and generate all neighbors of the OS string.

2) Randomly select one neighbor as the current OS string, denoted as $C$.

Also, adopting a job-based half mutation operator for the MS string, as shown in Fig. 3 (b). The half gene in each job will be mutated using a job-based half-mutation operator. After mutation operation, set $Gen=Gen+1$, and go to step 3.

***Step 7***: Output the global solution and feed it into ILS.

***Step 8***: The ILS algorithm explores the optimal local path for the JSSP and FJSSP instances. We set the maximum iteration to be 10, 000 and the maximum no-improved value for make-span is 0.02. The ILS algorithm used in this article is the same as our previous paper^8^.

***Step 9***: If some criteria are satisfied (generation step up to 10,000 or no improved value is less than 0.02) in the ILS algorithm, output the solution. The final solution will be decoded into JSSP or FJSSP pattern using reference^7^. Else, go to step 8.

## Experimental verification

The authors implemented the proposed method based on the system of Ubuntu 16.0.4 with Intel(R) i7-7700 CPU at 3.60 GHz, and the programming language is Python 3.5. Moreover, we compared the proposed method with some leading methods to indicate its effectiveness for both classical JSSP and FJSSP in some public instances.

### Verification for JSSP

#### Makespan

The JSSP is simpler than FJSSP as it does not require machine selection. We compared the proposed method with GA^39^, DDQN^4^, ACRL^35^, HDNN^31^, and ILS, GA1 (the GA in the proposed method) on some famous JSSP instances, including ft10 (10 $\times$ 10), ft20 (20 $\times 5$)^40^, la24 (15 $\times 10$), la36 (15 $\times 15$)^41^, abz7 (20 $\times$ 15)^42^, yn1(20 $\times$ 10)^43^. To fairly compare, the comparative results are collected directly from the original paper. The term '-’ means not report, and the term 'GA1' is the GA part of the proposed method. The comparative results in terms of makespan are shown in Table 2.

**Table 2:** The comparative results for solving JSSP in terms of makespan.

Method	ft10	ft20	la24	la36	abz7	yn1	Average scores
GA^39^	1203	1511	1336	1806	1050	1472	69.69
DDQN^4^	980	1208	1029	1355	725	996	92.74
ACRL^35^	1097	–	–	–	457	–	–
HDNN^31^	1023	1391	1056	1318	726	995	90.01
GA1	978	1187	971	1328	713	967	94.92
ILS	971	1181	984	1423	787	980	92.24
Proposed	**966**	**1178**	**950**	**1288**	**687**	**955**	**96.94**
Optimal	930	1165	935	1268	665	886	1.0

**Figure 4:**
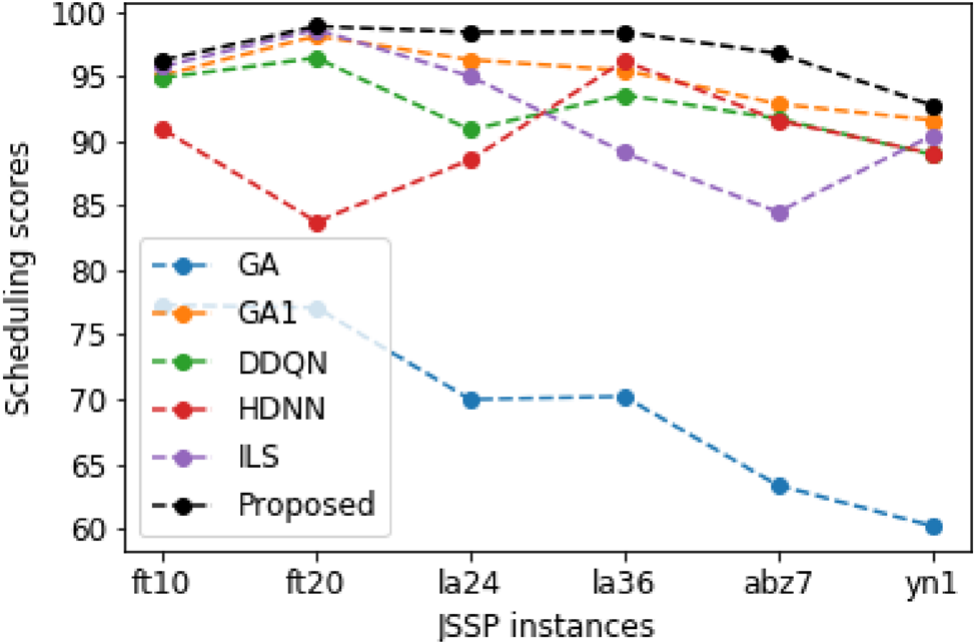
The scheduling scores for each method.

The results indicated that the proposed method outperforms others for all JSSP instances. Besides, the GA1 applied in the proposed method performs better than GA^39^. ILS performs better than GA1 on ft10 and ft20. We calculate the scheduling score $score=\frac{{C}_{optimal}}{{C}_{al}}$, as shown in Fig. 4, except method ACRL^35^ since it is incomplete. Where ${C}_{al}$ and ${C}_{Optimal}$ are the makespans of each method and optimal solution. The results indicated that the proposed method obtained scheduling scores higher than 90% for six JSSP instances and outperformed others. The averaged scheduling scores are also given in Table 2. The results confirmed the proposed method's effectiveness, with an average scheduling score of 96.94%. To show the priority of the proposed improved GA1, we compare it with GA^39^. The calculation result indicated that GA1 had improved a 36.20% = (94.92–69.69)/69.69 scheduling score compared to GA^39^. Also, the application of ILS has improved by 2.13% = (96.94–94.92)/94.92 of the average scheduling score, which is conducted by comparing the proposed method with GA1. The priority of each method could be ranked as: The proposed > GA1 > ILS > DDQN > HDNN > GA.

**Figure 5:**
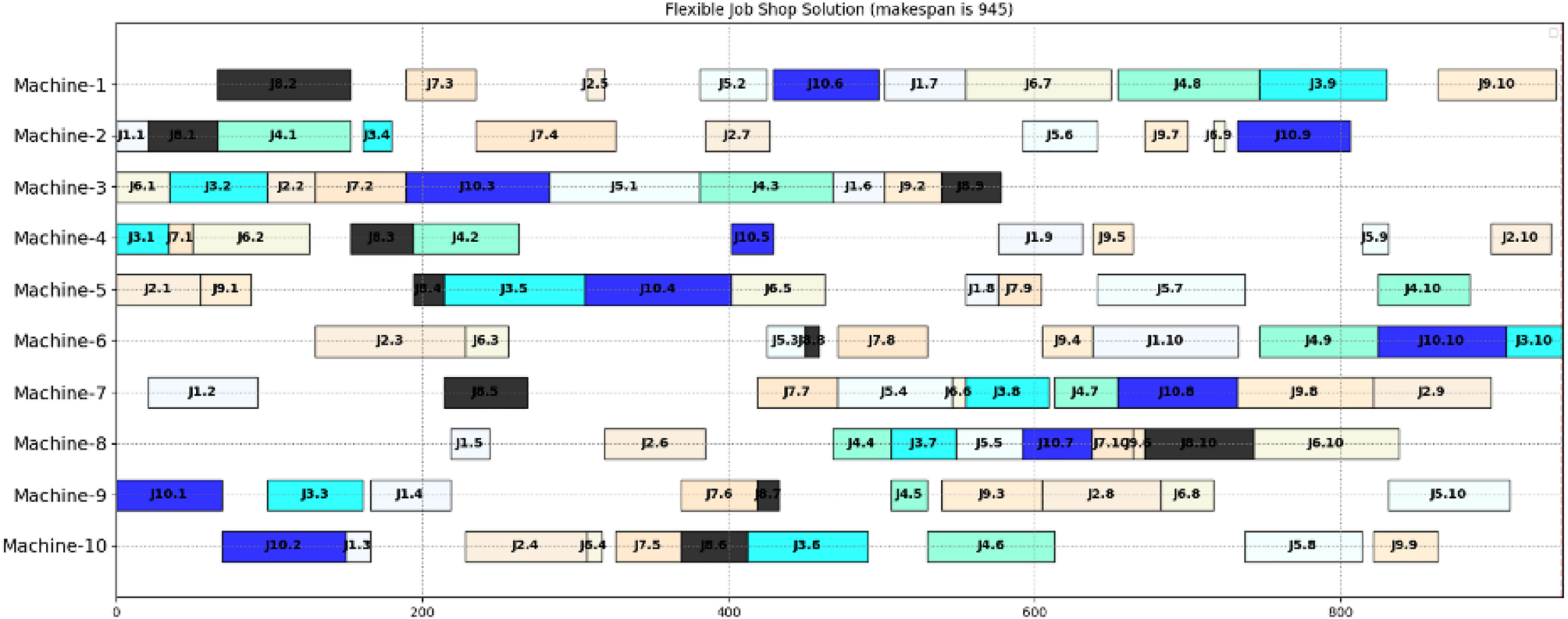
The solution of la16 with the proposed method.

We also compare the proposed method with the current leading learning-based methods, DDQN^4^, ACRL^35^, ML-CNN^8^, and GA1, ILS, on more la01 to la20^41^. The results in terms of makespan are shown in Table 3. The findings showed that the proposed method performs much better than ACRL^35^ on la11 to la 15, whose solutions are optimal, while ACRL^35^ is not for la12, la133, and la15. Besides, the proposed method won all cases for twenty JSSP instances compared to other algorithms. In addition, it received eighteen optimal solutions except for la20. It indicated that the proposed method outperforms others and could effectively solve JSSP in terms of makespan. The solution of la16 using the proposed method is shown in Fig. 5, whose makespan is 945.

**Table 3:** The comparative results for solving JSSP in terms of makespan.

	DDQN	ACRL^35^	ML-CNN	GA1	ILS	Proposed	Optimal	Score
la01(10 $\times$ 5)	**666**	**–**	**666**	**666**	**666**	**666**	666	**100**
la02(10 $\times$ 5)	**655**	**–**	**655**	671	667	**655**	655	**100**
la03(10 $\times$ 5)	**597**	**–**	603	620	617	**597**	597	**100**
la04(10 $\times$ 5)	609	**–**	**590**	602	**590**	**590**	590	**100**
la05(10 $\times$ 5)	**593**	**–**	**593**	**593**	**593**	**593**	593	**100**
la06(15 $\times$ 5)	**926**	**–**	**926**	**926**	**926**	**926**	926	**100**
la07(15 $\times$ 5)	**890**	**–**	**890**	**890**	**890**	**890**	890	**100**
la08(15 $\times$ 5)	**863**	**–**	**863**	**863**	**863**	**863**	863	**100**
la09(15 $\times$ 5)	**951**	**–**	**951**	**951**	**951**	**951**	951	**100**
la10(15 $\times$ 5)	**958**	–	**958**	**958**	**958**	**958**	958	**100**
la11(20 $\times$ 5)	**1222**	**1222**	**1222**	**1222**	**1222**	**1222**	1222	**100**
la12(20 $\times$ 5)	1047	1111	**1039**	**1039**	**1039**	**1039**	1039	**100**
la13(20 $\times$ 5)	1151	1181	**1150**	**1150**	**1150**	**1150**	1150	**100**
la14(20 $\times$ 5)	**1292**	**1292**	**1292**	**1292**	**1292**	**1292**	1292	**100**
la15(20 $\times$ 5)	1221	1288	**1207**	1209	**1207**	**1207**	1207	**100**
la16(10 $\times$ 10)	980	**–**	968	982	984	**945**	945	**100**
la17(10 $\times$ 10)	799	**–**	789	793	792	**784**	784	**100**
la18(10 $\times$ 10)	859	**–**	861	869	861	**848**	848	**100**
la19(10 $\times$ 10)	872	**–**	846	891	865	**842**	842	**100**
la20(10 $\times$ 10)	924	**–**	912	921	**907**	**907**	902	**99.45**
Best rankings	**11**	**–**	**14**	**11**	**14**	**20**	**–**	**20**

Significant values are in bold.

**Figure 6:**
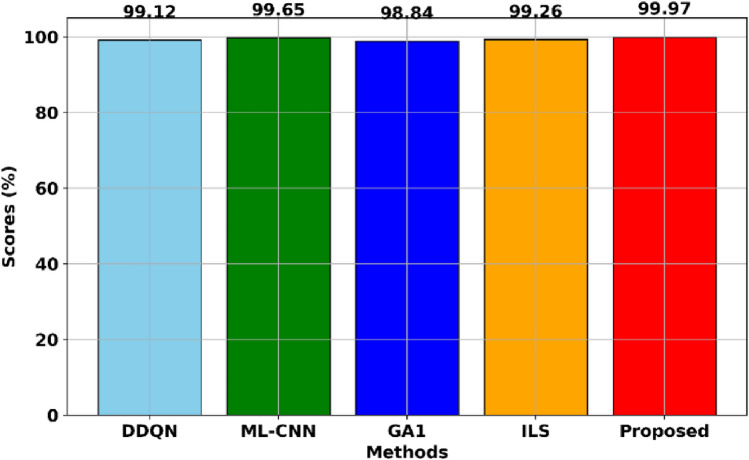
The average scheduling scores of each method for solving JSSP on la01-la20.

To see the difference between the proposed method and others, we calculate each method's average scheduling score except ACRL^35^, as shown in Fig. 6. The results indicated that the proposed method is the most near to the optimal method, whose average scheduling scores are 99.97%. Besides, all method's scheduling scores are near 99%, but the proposed method is the highest. According to the average scheduling scores, they can rank as: The proposed > ML-CNN > ILS > DDQN > ILS. In addition, the application of ILS has improved the scheduling scores from 98.84 to 99.97, proving that the GA lacks some local search capacity and that ILS could guide GA to find the best local path.

#### Average utilization rate (AUR)

**Figure 7:**
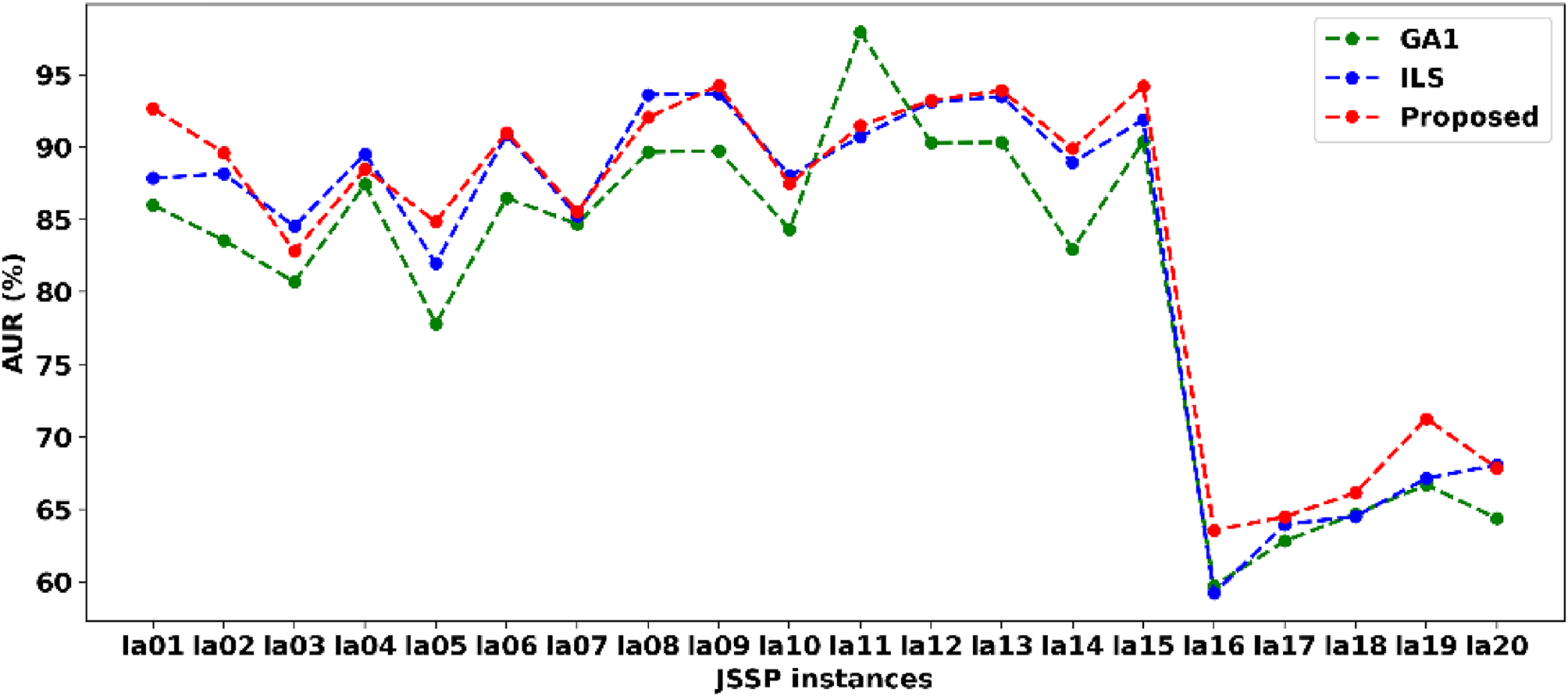
The average utilization ratio of each method for solving JSSP on la01-la20.

The proposed method aims at optimizing multiple objects, including makespan, AUR, and ML, for each instance. Here, we give the AUR for each JSSP instance using GA1, ILS, and the proposed method since all MLs are the same for JSSP, as shown in Fig. 7. The results showed that the proposed method won fourteen times out of twenty in terms of AUR, including la01, la02, la05, la06, la07, la09, and la12-la19. On the contrary, GA only won one time on la12, and ILS won five times out of twenty, including la03, la04, la08, la10, and la20, respectively. The results confirmed each component's effectiveness again in terms of AUR. In summary, the proposed method could process JSSP effectively within a satisfactory makespan and AUR.

### Verification for FJSSP

The above section has confirmed the effectiveness of the proposed method for solving JSSP. This section focuses on verifying its effectiveness for FJSSP. We compared the proposed method with existing leading methods, including TSN1 and TSN2^44^, the improved GA1, and ILS on forty FJSSP instances from rdata^44^.

#### Makespan

**Figure 8:**
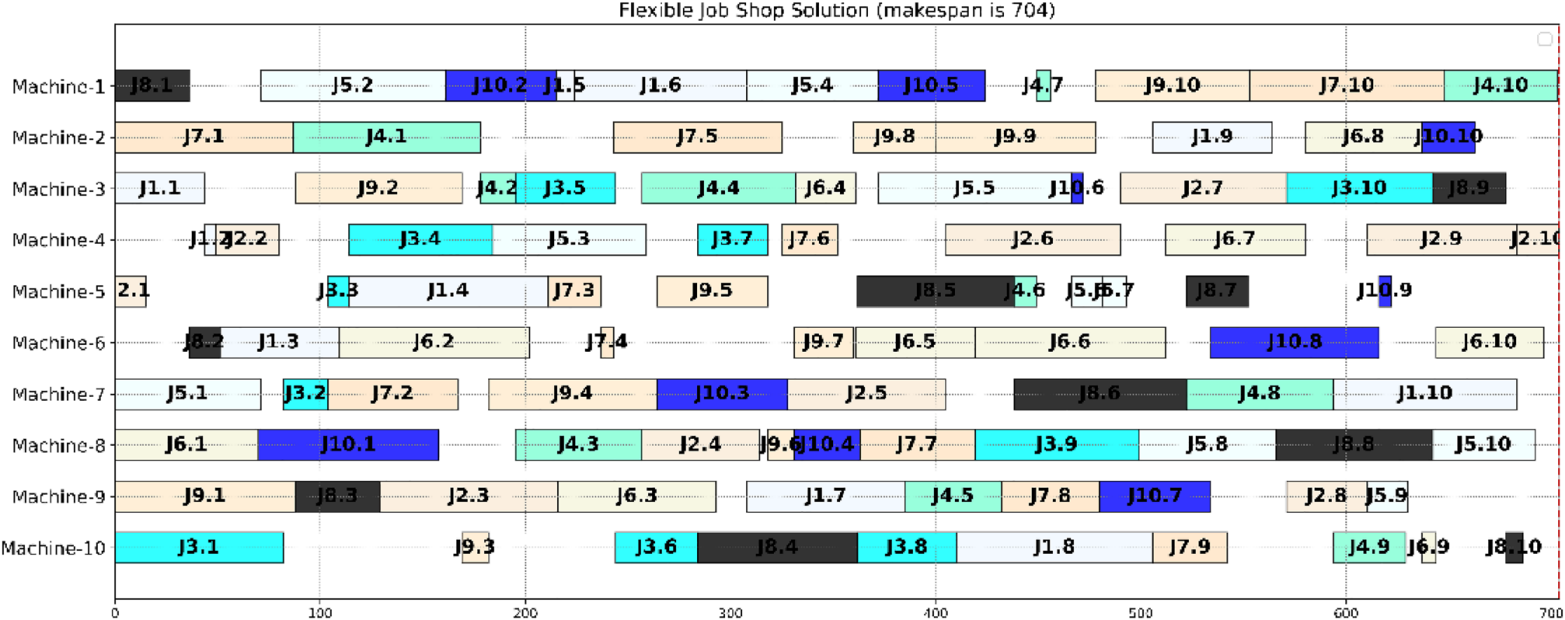
The FJSSP solution of la19 using the proposed, whose makespan is 704.

The comparative results in terms of makespan are shown in Table 4. The results indicated that the proposed method won thirty-seven times out of forty FJSSP instances in terms of makespan, while the components of GA1 and ILS won zero. It illustrates that only using GA1 or ILS cannot solve FJSSP well since GA lacks local exploring capacity while ILS lacks global searching capacity. Combining GA and ILS could solve FJSSP effectively. TSN1 and TSN2 won six and seven times out of forty FJSSP instances. Moreover, ILS generally performs better than GA1. Similar to makespan, the scheduling score results showed that the proposed method is near 99% and won 37 times out of 40 FJSSP instances. Those methods could be ranked as: The proposed > TSN2 > TSN1 > ILS > GA. One example of la19's solution using the proposed method is given in Fig. 8, whose makespan is 704.

**Table 4:** The comparative results for FJSSP in terms of makespan.

	GA1	ILS	TSN1	TSN2	Proposed	Optimal	Score
la01(10 $\times$ 5)	590	577	580	577	**573**	570	**99.48**
la02(10 $\times$ 5)	563	541	536	535	**532**	530	**99.62**
la03(10 $\times$ 5)	495	508	486	486	**480**	477	**99.38**
la04(10 $\times$ 5)	524	511	509	506	**504**	502	**99.60**
la05(10 $\times$ 5)	461	464	464	**458**	**458**	457	**99.78**
la06(15 $\times$ 5)	805	802	804	803	**800**	799	**99.88**
la07(15 $\times$ 5)	766	754	754	752	**751**	749	**99.73**
la08(15 $\times$ 5)	787	770	767	768	**766**	765	**99.87**
la09(15 $\times$ 5)	860	860	859	857	**854**	853	**99.88**
la10(15 $\times$ 5)	809	809	806	**805**	**805**	804	**99.88**
la11(20 $\times$ 5)	1097	1077	1073	1073	**1072**	1071	**99.91**
la12(20 $\times$ 5)	1057	939	937	**937**	**937**	936	**99.89**
la13(20 $\times$ 5)	1067	1041	1039	1039	**1038**	1038	**100**
la14(20 $\times$ 5)	1092	1072	1071	1071	**1070**	1070	**100**
la15(20 $\times$ 5)	1114	1092	1093	1093	**1091**	1090	**99.91**
la16(10 $\times$ 10)	804	741	**717**	**717**	**717**	717	**100**
la17(10 $\times$ 10)	652	646	**646**	**646**	**646**	646	**100**
la18(10 $\times$ 10)	709	704	674	673	**669**	666	**99.55**
la19(10 $\times$ 10)	769	761	725	709	**704**	700	**99.43**
la20(10 $\times$ 10)	814	761	**756**	**756**	**756**	756	**100**
la21(15 $\times$ 10)	916	939	861	861	**859**	835	**97.21**
la22(15 $\times$ 10)	843	842	790	795	**786**	760	**96.69**
la23(15 $\times$ 10)	932	945	884	887	**859**	840	**97.79**
la24(15 $\times$ 10)	878	898	**825**	830	830	806	97.11
la25(15 $\times$ 10)	881	871	823	821	**808**	789	**97.65**
la26(20 $\times$ 10)	1169	1162	1086	1087	**1076**	1061	**98.61**
la27(20 $\times$ 10)	1201	1179	1109	1115	**1102**	1089	**98.82**
la28(20 $\times$ 10))	1164	1155	1097	**1090**	**1090**	1079	**98.99**
la29(20 $\times$ 10)	1087	1079	1016	1017	**1008**	997	**98.91**
la30(20 $\times$ 10)	1180	1163	1105	1108	**1103**	1078	**97.73**
la31(30 $\times$ 10)	1633	1567	1532	1533	**1526**	1521	**99.67**
la32(30 $\times$ 10)	1764	1704	1668	1668	**1661**	1659	**99.88**
la33(30 $\times$ 10)	1607	1543	1511	1507	**1503**	1499	**99.73**
la34(30 $\times$ 10)	1639	1565	1542	1543	**1539**	1536	**99.81**
la35(30 $\times$ 10)	1646	1602	1559	1559	**1554**	1550	**99.74**
la36(15 $\times$ 15)	1146	1189	**1054**	1071	1060	1028	96.98
la37(15 $\times$ 15)	1218	1211	1122	1132	**1108**	1074	**96.93**
la38(15 $\times$ 15)	1105	1110	1004	1001	**989**	960	**97.07**
la39(15 $\times$ 15)	1165	1151	**1041**	1068	1054	1024	97.15
la40(15 $\times$ 15)	1120	1113	1009	1009	**994**	970	**97.59**
Best rankings	0	0	**6**	**7**	**37**	**–**	**37**

Significant values are in bold.

**Figure 9:**
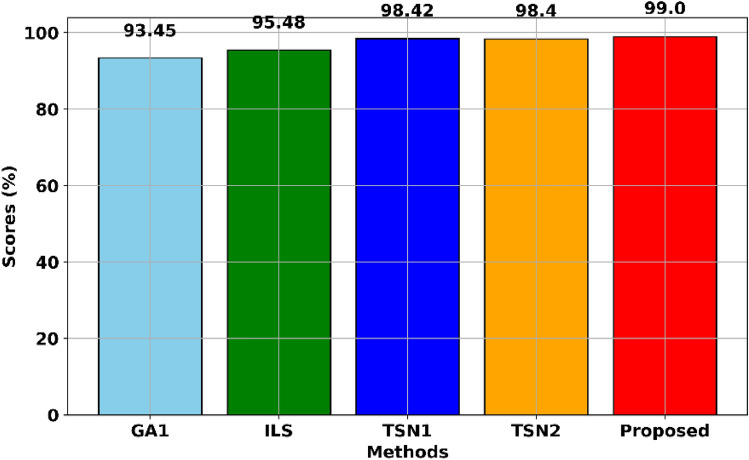
The average scheduling scores of each method for solving FJSSP on la01-la40.

To see each method's difference, we calculate the average scheduling scores of each method, as shown in Fig. 9. The results confirmed that the proposed method is one near-optimal method for solving FJSSP, whose average scheduling score is 99.0%. Another finding is that only using GA1 or ILS cannot solve FJSSP well, as their average scheduling scores are 93.45% and 95.48%, which are far from the proposed method. The performance of each method for solving FJSSP could be ranked as: The proposed > TSN1 > TSN2 > ILS > GA1 according to average scheduling scores.

#### Average utilization ratio (AUR)

**Figure 10:**
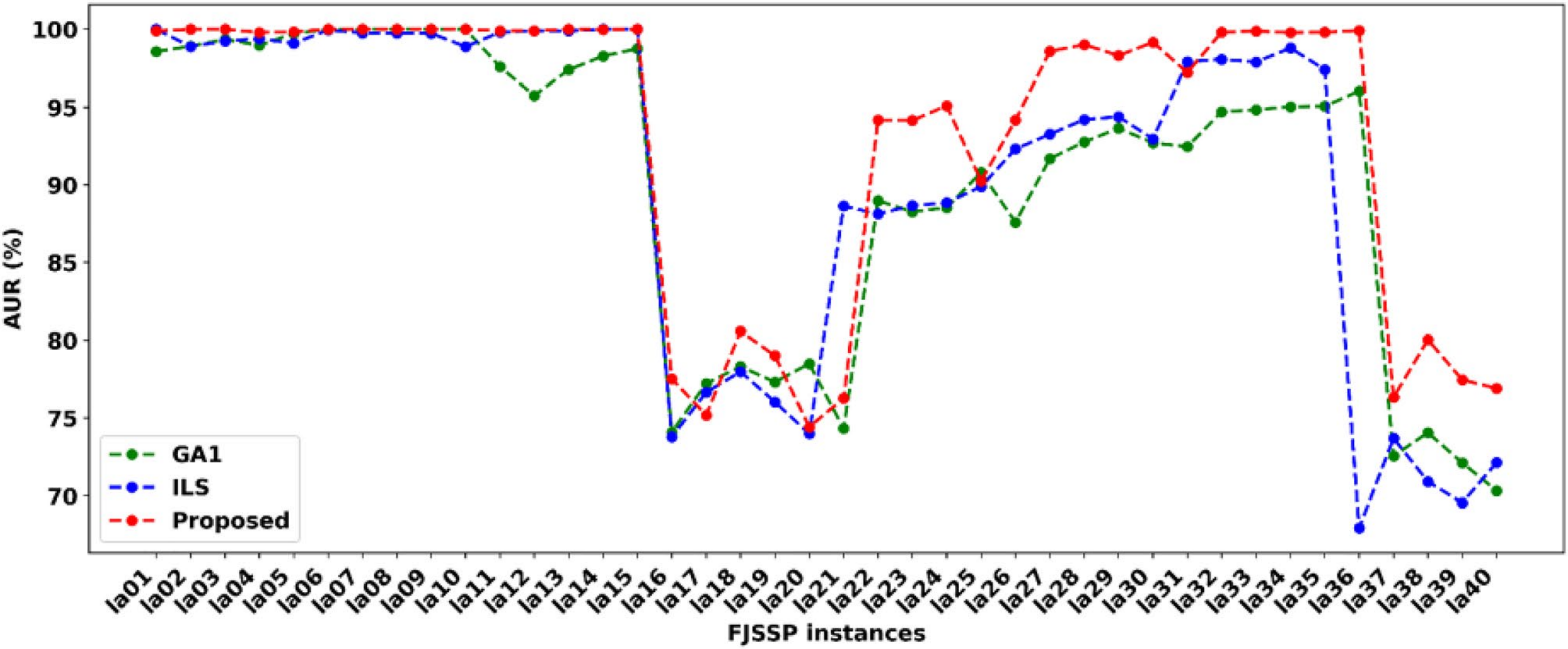
The AUR of each method for solving FJSSP on la01-la40.

The AUR illustrates each machine's utilization ratio during the whole production process. Generally, the higher the AUR, the better performance the algorithm shows. We calculate the AUR of each method on forty FJSSP, as shown in Fig. 10. The results showed that the proposed method performed better and won most cases. It won thirty-three times out of 40 FJSPP instances, including la02-la13, la15, la16, la18, la19, la22-la24, la26-la30, and la32-la40, respectively. On the contrary, GA1 and ILS only won eight and six times out of forty. The proposed method has an absolute advantage on large-scale FJSSP instances conducted from la23-la40. Moreover, all methods perform well on small-scale FJSSP instances, from la01 to la16, whose AURs are high up to 99%. The above findings indicated that the proposed method could arrange each sub-operation well on the selected machine to execute for solving FJSSP, and only using GA or ILS cannot obtain satisfactory AUR.

#### Max loading (ML)

**Figure 11:**
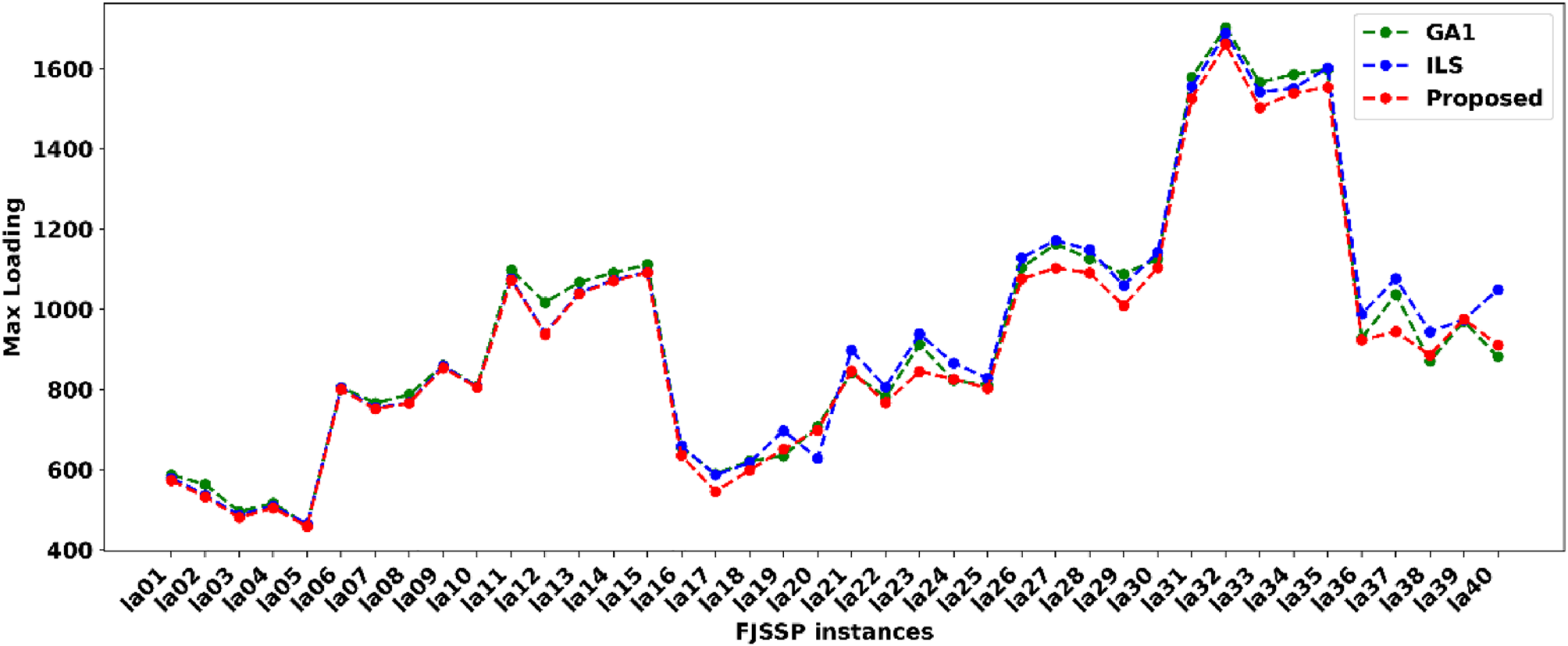
The ML of each method for solving FJSSP on la01-la40.

The max loading (ML) represents each machine's loading capacity. The huge load may damage the machine and result in a delay in the whole manufacturing process. Therefore, testing the proposed method's ML for each FJSSP instance is necessary. The results showed that the proposed method has a low ML compared to GA1 and ILS, as shown in Fig. 11. The proposed method won thirty-three times out of forty instances, including la01-la18, la22, la23, and la25-la37, respectively. However, GA1 only won six times, including la19, la21, la24, la38, la39, and la 40, respectively; and ILS only won la20. The above evidence showed that the proposed method could deal with FJSSP with low max loading, and only using GA1 or ILS cannot find the best solution due to the lack of local exploring and global searching capacities.

In summary, the proposed method could process FJSSP instances within satisfactory makespan, AUR, and ML due to its excellent global search and local exploring capacities.

## Discussion

Job shop scheduling is critical in building one smart factory regarding resource supply and intelligent production. Most current methods only focus on one type of job shop scheduling: JSSP or FJSSP, and single-object, which cannot satisfy human beings' needs. This article proposes an effective scheduler, GAILS, to solve JSSP and FJSSP with multiple objects, including makespan, AUR, and ML.

Considering each algorithm's advantages and disadvantages, as summarized in Table 1. The proposed GAILS applied GA to find the global path and guide ILS to explore the optimal local path. In GA, three improved operators, including selection, crossover, and mutation operators, are utilized to find the best chromosome for each instance. Therefore, the proposed method has an excellent search capacity and could balance globality and diversity. The whole process of the proposed method includes nine steps, as shown in Fig. 1. Steps 1–6 are to find the global path, while steps 7–9 are for best local path exploring.

To verify the proposed method's effectiveness, we tested and compared the proposed method and several leading methods based on sixty-six JSSP instances. We compared the proposed method with GA^39^, DDQN^4^, ACRL^35^, HDNN^31^, and ML-CNN^8^ on twenty-six JSSP instances to validate its effectiveness for solving classical JSSP. The results in terms of makespan showed that the proposed method outperforms others, which can be seen in Tables 2 and 3. Besides, we calculated their scheduling scores to see their performance compared to the optimal method. The results indicated that the proposed method is near the optimal method, whose average scheduling score is higher than 95%, which could be conducted in Figs. 4 and 6.

To verify each component's effectiveness for solving JSSP in the proposed method, we compared it with GA1 and ILS. The results showed that only using GA1 or ILS cannot solve JSSP effectively, as shown in Tables 2, 3, and Figs. 4 and 6. One solution using the proposed method for solving la16 is given in Fig. 5. The AUR testing results indicated that the proposed method could deal well with JSSP with a good AUR, as shown in Fig. 7.

Moreover, we tested and compared the proposed GAILS with TSN1 and TSN2^44^ on forty FJSSP instances to validate its effectiveness. The results in terms of makespan indicated that the proposed method performs the best. It won thirty-seven times out of forty, as shown in Table 4. Similar to JSSP, we calculated the scheduling score of the proposed method, and the results showed that the proposed method could receive 99.0% of the average scheduling score for solving FJSSP, as shown in Fig. 9. It indicated that the proposed method is one near-optimal solution for FJSSP. Besides the comparative results between the proposed GAILS and GA1, ILS has confirmed each component's effectiveness. Another finding is that using GA1 or ILS alone cannot solve FJSSP well, as their average scheduling scores are 93.45% and 95.48%, which are far from the proposed method. One solution using the proposed method based on the la19 FJSSP instance is given in Fig. 8, whose makespan is 704.

The AUR results showed that the proposed method could deal with FJSSP instances well with satisfactory AUR, as shown in Fig. 10. The proposed method has an absolute advantage on large-scale FJSSP instances.

The ML testing results showed that the proposed method could arrange each operation on the selected machine well and has a small ML, which could be conducted in Fig. 11. The comparative results between the proposed method and GA1, ILS, confirmed the proposed method's effectiveness again.

In summary, the proposed method could effectively process both JSSP and FJSSP within multiple objectives. By changing the order of makespan, AUR, and ML in the fitness function, we can easily receive multiple solutions for different production statuses. However, GA is much more time-consuming as it requires executing the selection, crossover, and mutation operations 400 hundred times. Respectively, it takes almost half-day to process la40 while ILS only needs ten seconds. How to design one effective and time-saving GA to search global path is worth thinking more about.

## Conclusions

This article proposes an effective job shop scheduler, GAILS, to solve both JSSP and FJSSP within multiple objects, including makespan, AUR, and ML. In the proposed method, GA is used to search approximate global solutions, and the searched solution is fed into ILS to explore the best optimal local solution. In the GA, three improved operators, including selection, crossover, and mutation, are designed to select the best chromosome for the global solution by using one combination fitness function made of makespan, AUR, and ML. Thus, the proposed method has an excellent searching capacity and could balance globality and diversity. The comparative analysis based on sixty-six instances has confirmed the effectiveness of the proposed method for both JSSP and FJSSP. It received a 96.94% average scheduling score in large-scale JSSP instances (see Table 2), won all cases on la01-la20 JSSP instances in terms of makespan, and won fourteen times out of twenty in terms of AUR. For FJSSP, it won thirty-seven, thirty-three, and thirty-three times out of forty FJSSP instances in terms of makespan, AUR, and ML, respectively.

As discussed in the above section, the GA used in the proposed method is much more time-consuming. In the future, we will focus on designing one time-saving GA to combine with ILS for solving JSSP and FJSSP. Besides, we will compare the proposed method with other multi-objective JSSP algorithms to validate its effectiveness.

## Data Availability

The datasets used in this article are from the well-known public dataset, which has been appropriately cited.
